# Supplementary material for: Multiple Posterior Insula Projections to the Brainstem Descending Pain Modulatory System
Source: Int J Mol Sci. 2024 Aug 24;25(17):9185. doi: 10.3390/ijms25179185 (PMC11395413; doi:10.3390/ijms25179185)
Supplement: Supplementary file 1 [file ijms-25-09185-s001.zip › ijms-3109590-supplementary.pdf]

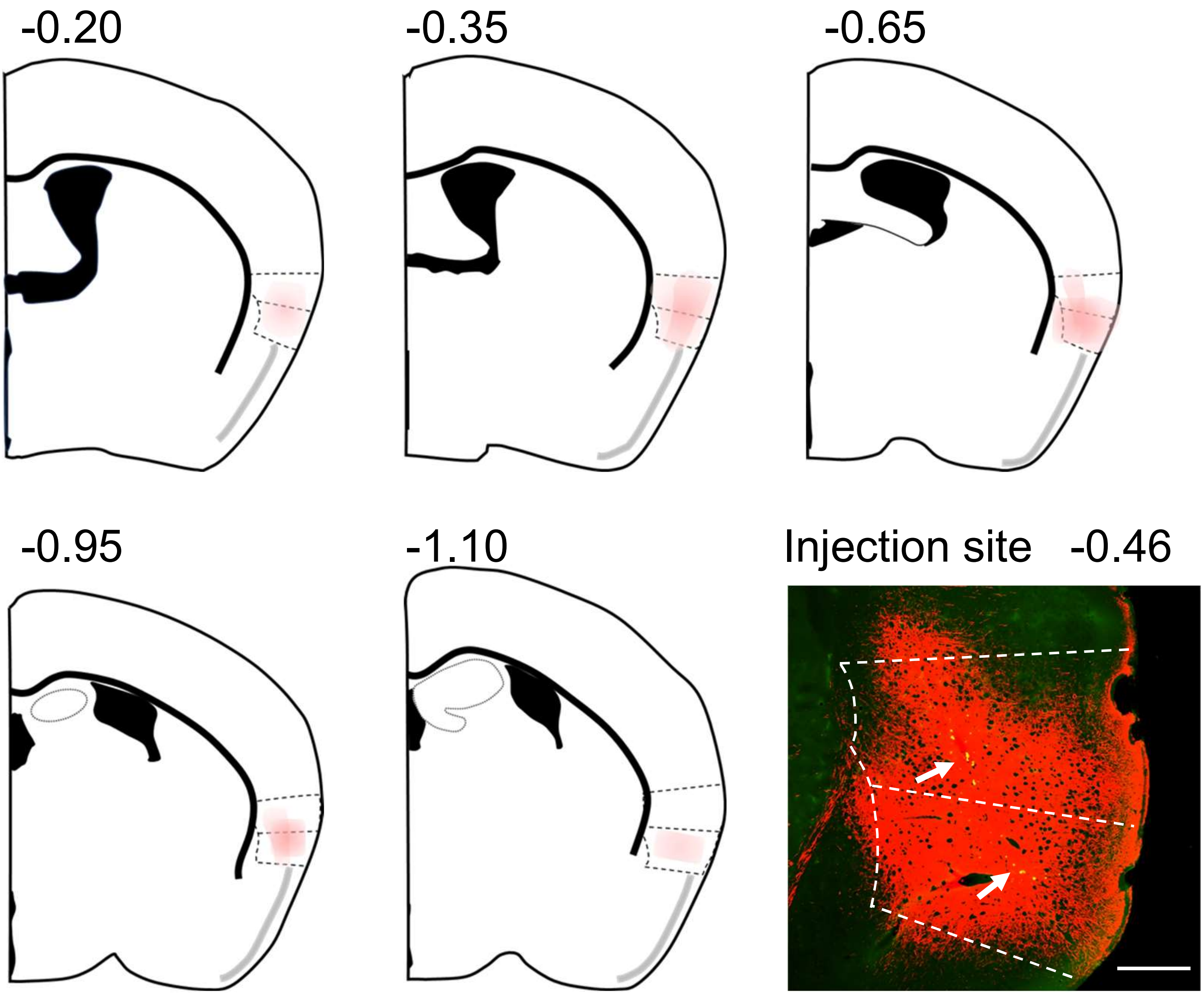

Supplementary figure S1. mCherry expression in the posterior Insula of animal DL12. Coronal schematic diagrams showing the extend of mCherry expression at different anterior-posterior levels. Numbers denote distance from Bregma (in cm). Image (right down) of the mCherry expression at the injection site (scale bar: 250 μm). Arrows show scar tissue from the injection syringe needle tract.

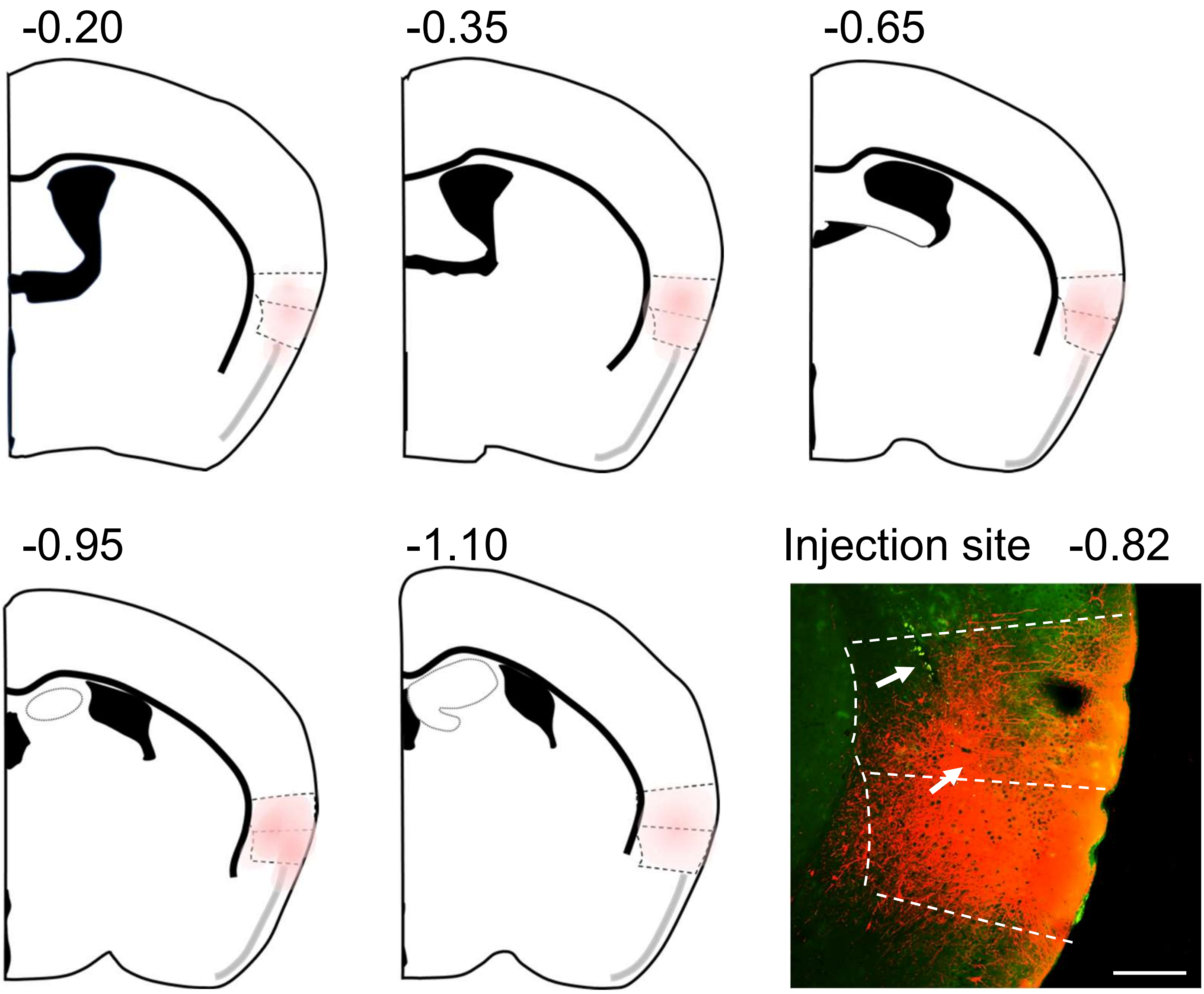

Supplementary figure S2. mCherry expression in the posterior Insula of animal DL17. Coronal schematic diagrams showing the extend of mCherry expression at different anterior-posterior levels. Numbers denote distance from Bregma (in cm). Image (right down) of the mCherry expression at the injection site (scale bar: 250 μm). Arrows show scar tissue from the injection syringe needle tract.

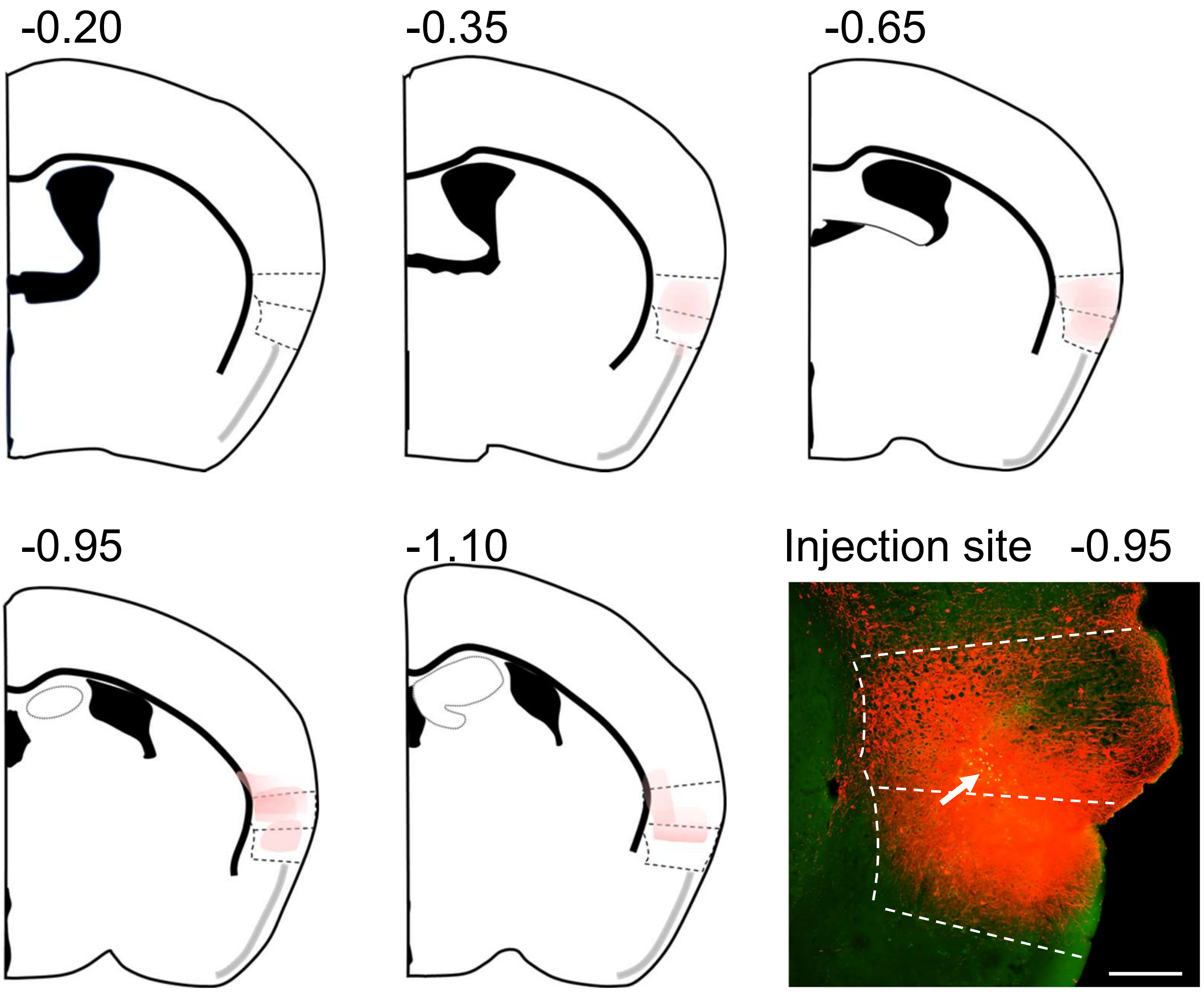

Supplementary figure S3. mCherry expression in the posterior Insula of animal DL27. Coronal schematic diagrams showing the extend of mCherry expression at different anterior-posterior levels. Numbers denote distance from Bregma (in cm). Image (right down) of the mCherry expression at the injection site (scale bar: 250 µm). The arrow shows scar tissue from the injection syringe needle tract.

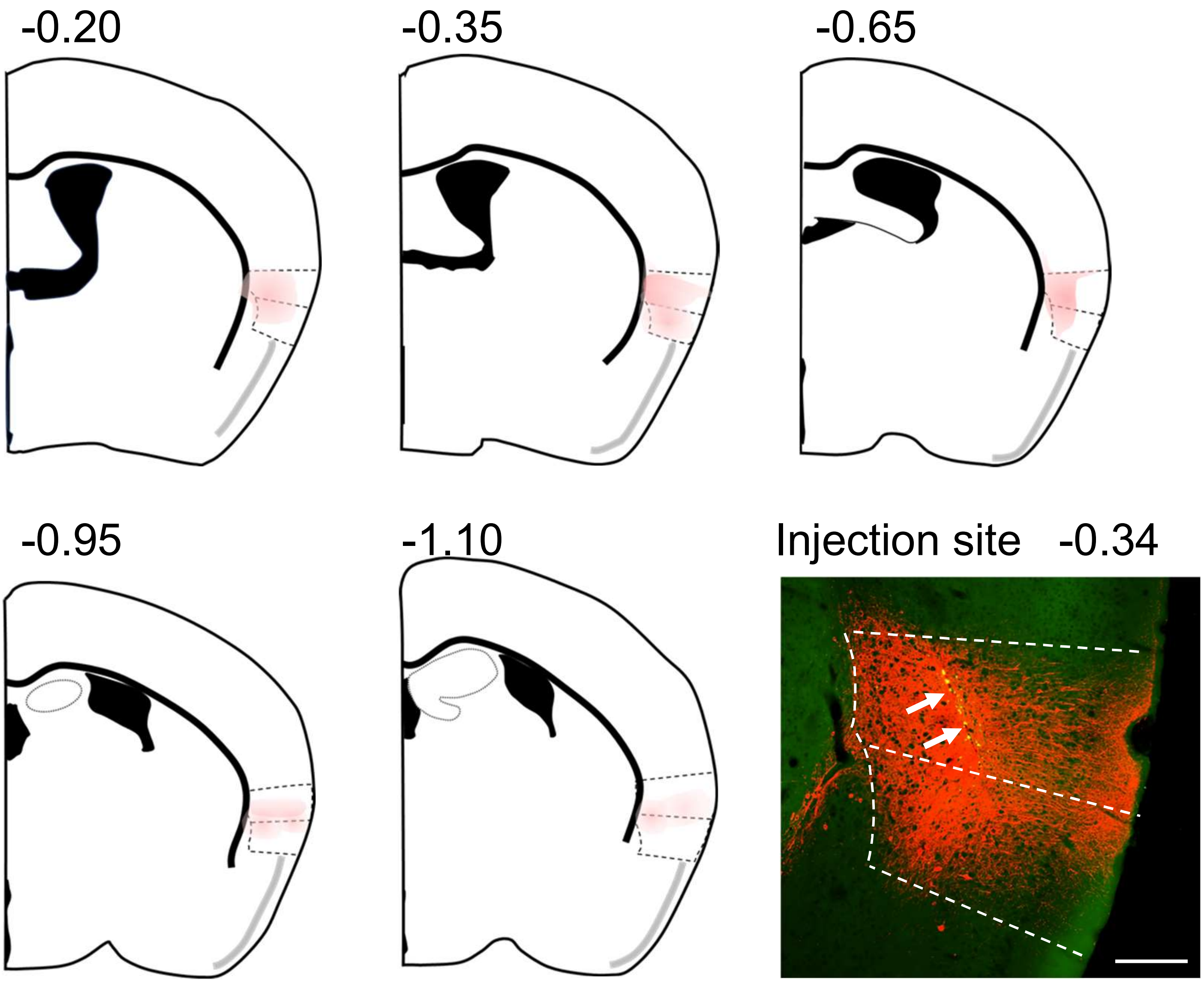

Supplementary figure S4. mCherry expression in the posterior Insula of animal DL28. Coronal schematic diagrams showing the extend of mCherry expression at different anterior-posterior levels. Numbers denote distance from Bregma (in cm). Image (right down) of the mCherry expression at the injection site (scale bar: 250 µm). Arrows show scar tissue from the injection syringe needle tract.

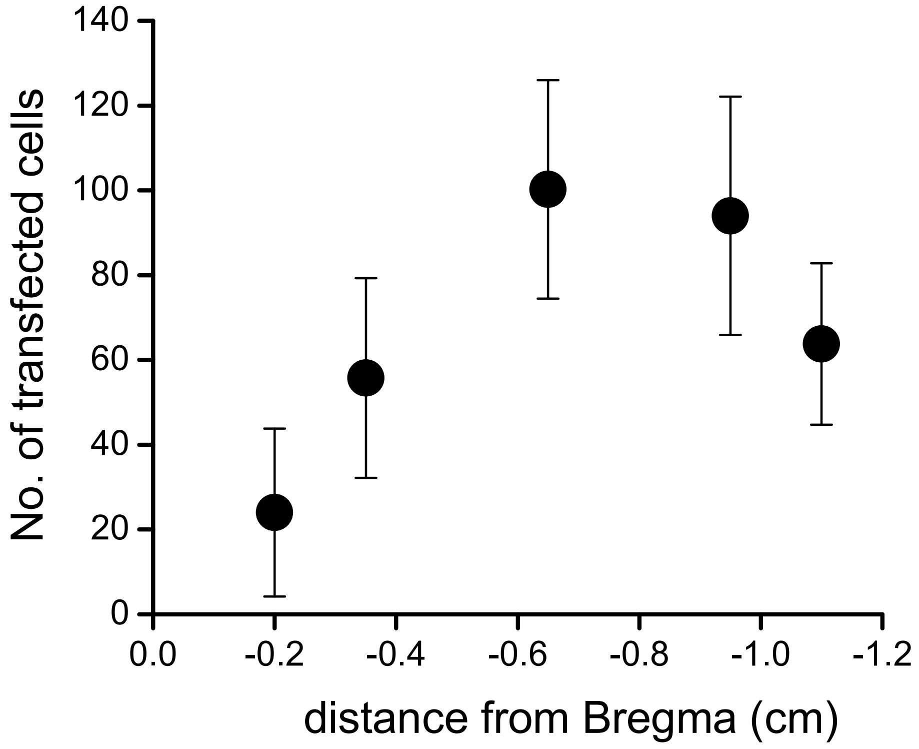

Supplementary figure S5. Viral transfection and mCherry expression in cell bodies along the rostro-caudal axis of the posterior insula. The number of mCherry positive somata of sections at different levels from Bregma spanning the rostro-caudal axis of the posterior insula were counted for each animal and the means ± SEM are shown (n = 4). The mean viral transfection maximum was between Bregma -0.6 and -0.8 close to the targeted injection site.

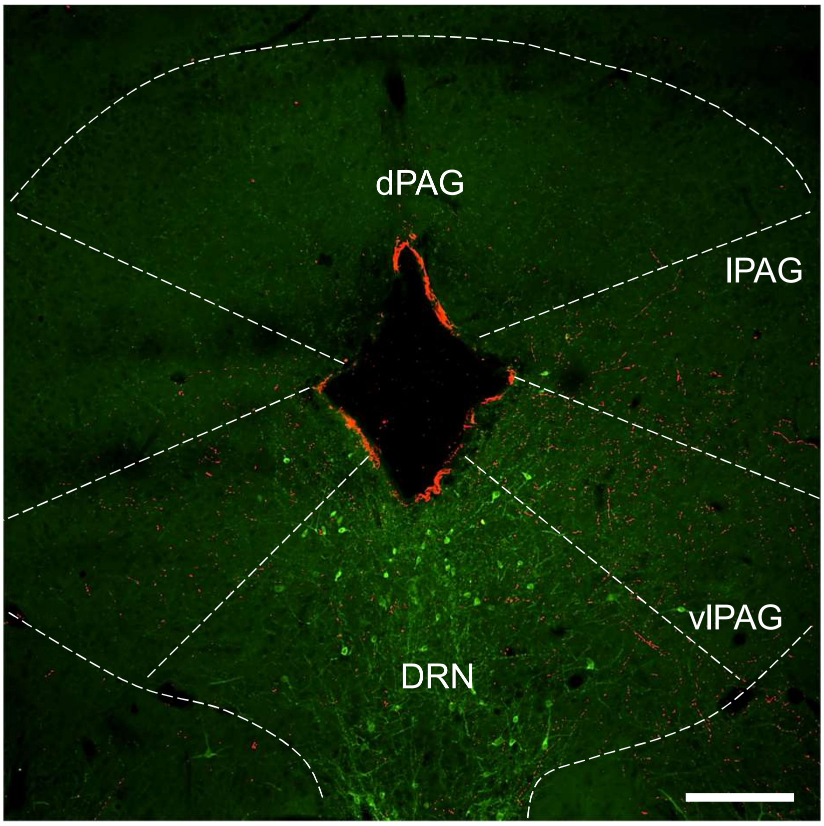

Supplementary figure S6. Low magnification image of the insular projections to the PAG. Immunostaining against mCherry (red) and against TH (green) of coronal sections containing the PAG after injection of viral vector carrying the mCherry gene in the posterior insula. The ipsilateral (right) and the contralateral (left) sides are shown, and the different areas of the PAG are delineated (dashed lines). dPAG: dorsal PAG (includes the dorsolateral and dorsomedial parts), DRN: Dorsal Raphe Nucleus, lPAG/vlPAG: lateral and ventrolateral parts of the PAG. Scale bar: 250 µm

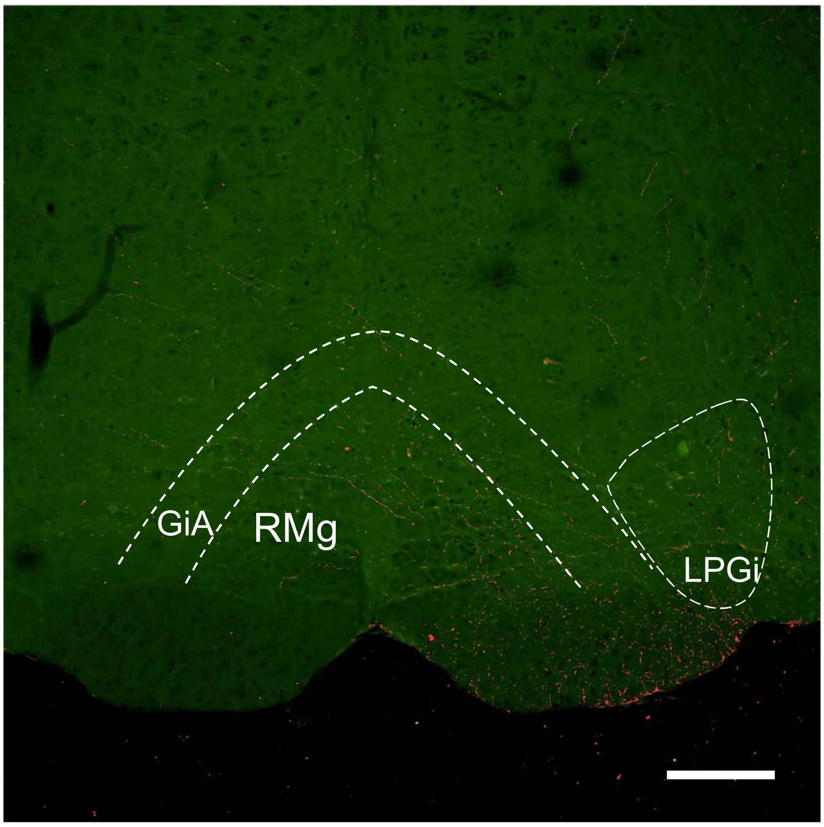

Supplementary figure S7. Low magnification image of the insular projections to the RVM. Immunostaining against mCherry (red) and against TH (green) of coronal sections containing the RVM after injection of viral vector carrying the mCherry gene in the posterior insula. The ipsilateral (right) and the contralateral (left) sides are shown and the different areas of the RVM, the gigantocellular reticular nucleus pars α (GiA) and RMg are delineated (dashed lines). LPGi: latral paragigantocellular nucleus. Scale bar: 250 μm

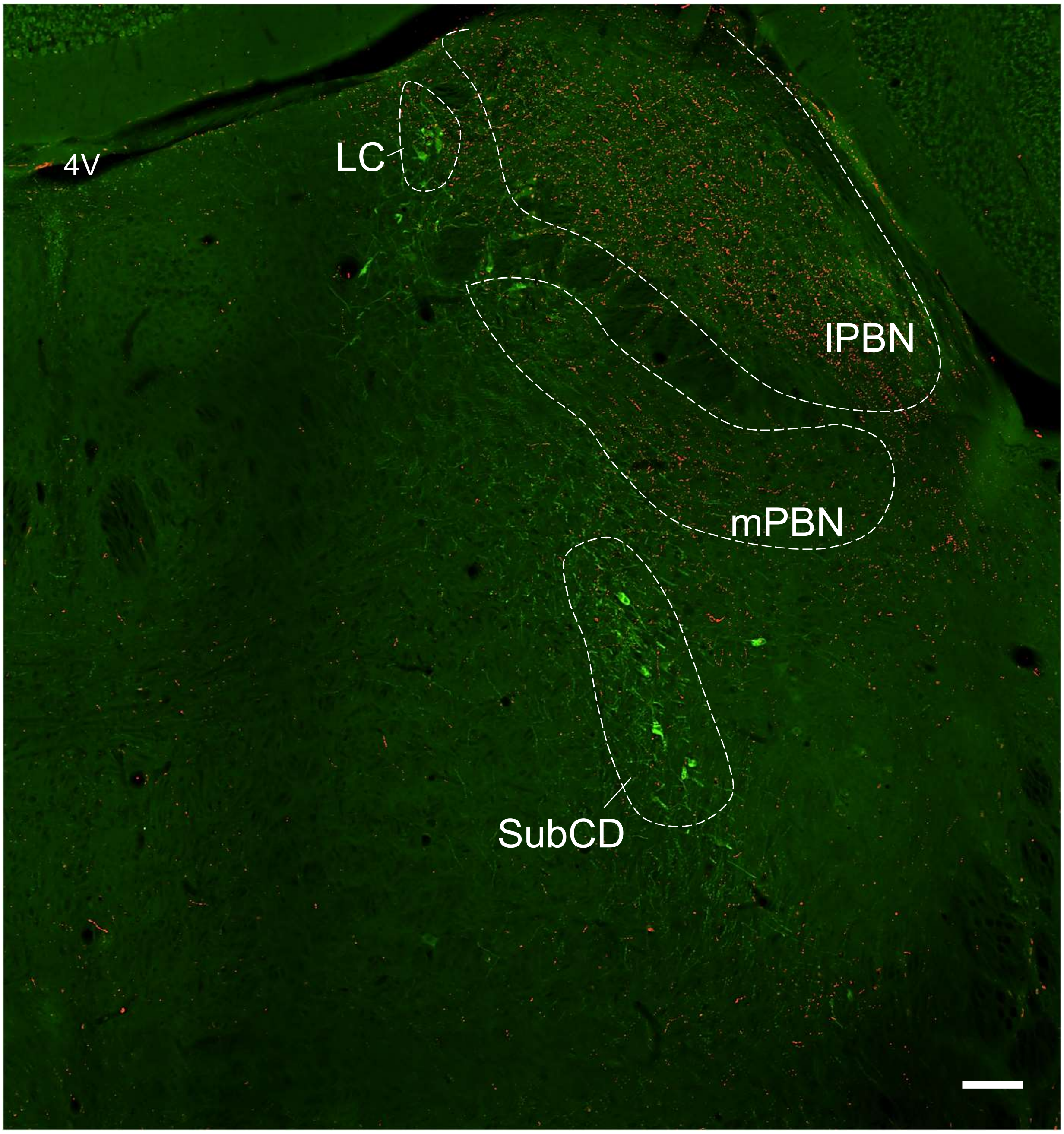

Supplementary figure S8. Mesoscale image of insula projections to different pontine nuclei. Immunostaining against mCherry (red) and against TH (green) of coronal sections containing the LC, lPBN, mPBN and SubCD (dased lines) after injection of viral vector carrying the mCherry gene in the posterior insula is shown. Only the ipsilateral side is shown. The image also exhibits our approach for distinguishing between LC and SubCD, with the LC identified close to the 4th ventricle (4V) while the SubCD at a distance ventrally from the ventricle. Scale bar: 150µm.
